# Master’s programs in vaccinology in Spain: a nationwide systematic environmental scan and a Delphi-informed core curriculum proposal

**DOI:** 10.3389/fpubh.2026.1707015

**Published:** 2026-02-02

**Authors:** Abelardo Claudio Fernández-Chávez, Jesús María Aranaz Andrés, Daniel Leonardo Sánchez-Carmona, Cristina Hernán-García, Fernando de Jesús Franco

**Affiliations:** 1 Department of Preventive Medicine and Public Health, Ramón y Cajal University Hospital, Madrid, Spain; 2 Ramón y Cajal Institute for Health Research (IRYCIS), Madrid, Spain; 3 Preventive Medicine and Public Health Service, Hospital Clínico Universitario de Valladolid, Valladolid, Spain; 4 Facultad de Ciencias de la Salud Universidad Internacional de la Rioja, Logroño, Spain

**Keywords:** vaccinology education, master’s programs, environmental scan, curriculum development, Delphi consensus

## Abstract

**Introduction:**

Vaccination is a cornerstone of public health; however, the master’s-level training in Spain remains fragmented and largely delivered through non-official master’s degrees (Spanish *título propio*) with heterogeneous structures and uneven coverage of critical domains. The lack of harmonized competency standards limits workforce readiness and international comparability. This study aimed to map the national training landscape and develop a consensus-based, competency-oriented core curriculum for a future official master’s program in vaccinology.

**Methods:**

We conducted a nationwide systematic environmental scan (May–June 2025) of master’s programs using official registries (RUCT, ANECA) and university websites. Eligible programs were required to be active in 2025–2026 and to have a publicly available syllabus. Two reviewers independently screened records and extracted data on European Credit Transfer and Accumulation System (ECTS) credits, delivery format, practicum and thesis requirements, tuition, language, and stated competencies. We then conducted a modified Delphi with a multidisciplinary expert panel. Consensus on the final domains was defined as a mean score of ≥4 (on a Likert scale of 1–5) and ≥80% agreement among participants. The agreed domains and their proposed ECTS allocations were compiled into a core curriculum worth 60 ECTS.

**Results:**

Of 20 records identified, 7 programs met the inclusion criteria. Only one—the Erasmus Mundus LIVE (Leading International Vaccinology Education)—was an official master’s degree (state-regulated, RUCT/ANECA-accredited; 120 ECTS); the remaining programs were university-awarded, non-official master’s degrees (24–60 ECTS). The majority of these programs were delivered online, primarily in Spanish; total tuition ranged from €560 to €10,159. All programs covered scientific fundamentals, epidemiology, and translational research; however, the inclusion of topics such as regulation/pharmacovigilance, health economics, risk communication, vaccine confidence, special populations, and internships was inconsistent. Four programs required a master’s thesis; only one offered professional internships (LIVE; 3 ECTS). The Delphi process produced an eight-domain framework with competency-based learning outcomes and 60 ECTS distributed across domains.

**Discussion:**

Spain’s master’s-level vaccinology education is heterogeneous, with gaps in regulatory science, economics, cross-cutting competencies (leadership, communication, ethics, and equity), special-population vaccination, and experiential training. A shared, competency-based framework can guide program redesign, enhance transparency for learners and employers, and support quality assurance and international alignment.

**Conclusion:**

We propose a Delphi-informed, eight-domain, 60-ECTS core curriculum to harmonize master’s-level vaccinology education in Spain, strengthen workforce preparedness, and align national training with international expectations.

## 1 Introduction

Vaccinating healthy people to prevent disease reflects one of the fundamental principles of preventive medicine (1). The expanded program on immunization (EPI) against 14 pathogens promoted by the World Health Organization (WHO) has prevented approximately 154 million deaths in the last 50 years, of which 146 million were children under 5 years of age. A child born today has a 40% higher survival rate for each year of infancy and childhood. The survival benefits of childhood vaccination extend beyond age 50, a remarkable finding given the exclusion of smallpox vaccination and the absence of expected benefits from vaccination against human papillomavirus (HPV), influenza, severe acute respiratory syndrome coronavirus 2 (SARS-CoV-2), Ebola, mpox, and other vaccines that affect adult mortality (2).

There has been dramatic growth in vaccinology courses since 2000, with nearly 9,500 professionals trained in face-to-face courses and 9,800 in online courses in Spain by 2018, a figure that increased significantly by 2022 in several regions. Alumni of these programs have taken on leadership roles and established post-course networks and activities (3). Currently, there is at least one specialization course in each WHO region (4).

However, significant challenges still remain. The lack of sustainable funding is a primary concern, with very little government support (5). There are geographical gaps, and the majority of courses are taught in English, which excludes many non-English speakers (6). The coronavirus disease of 2019 (COVID-19) pandemic forced courses to be postponed or adapted to hybrid or online formats. A web portal has been created to centralize course information^1^, and it is recommended that languages be diversified and hybrid formats be encouraged to expand access.

Vaccinology has been defined by the Royal National Academy of Medicine as a scientific discipline and a branch of therapeutics focused on the comprehensive study of vaccines, including their development, research, efficacy, effectiveness, and the epidemiology of vaccine-preventable diseases (7). However, this definition is often considered insufficient as it limits the understanding of vaccinology to a biomedical perspective and neglects its preventive and social dimensions (8). In practice, vaccinology is a multidisciplinary science that integrates knowledge of immunology, microbiology, epidemiology, public health, statistics, and regulatory and logistical areas and plays a strategic role in contemporary global health (9).

In Spanish medical schools, the teaching of vaccination accounts for relatively few hours or credits and is usually addressed only sporadically within courses such as pediatrics, microbiology, or preventive medicine, without in-depth coverage of vaccinology (10).

A global review of undergraduate health sciences education reveals that vaccinology content is typically incorporated in a fragmented and heterogeneous manner across countries. In various regions, including the United States, Europe, Latin America, and parts of Asia, the topic of vaccines is primarily covered within core subjects (e.g., microbiology, public health, and pediatrics), rather than as standalone, compulsory modules (11, 12). Evidence indicates that students and professionals frequently report insufficient training in vaccinology, particularly regarding communication with vaccine-hesitant patients, understanding regulatory frameworks, and the development of practical skills (13). Recent initiatives in countries such as the US, Japan, and Mexico have begun to address these gaps through elective courses or national curriculum frameworks (12, 14, 15), underscoring the international need for more systematic, integrated, and practice-oriented vaccinology education.

The absence of a unified training framework in vaccinology has prompted professional bodies such as the Spanish Association of Vaccinology (AEV) to call for the creation of a dedicated course within the undergraduate curricula of medicine, nursing, and pharmacy (16).

At the level of medical specialties, Spain does not have an official specialty in “vaccinology” nor a standardized training pathway within the national residency system (MIR). Training in vaccinology is fragmented across specialties, including preventive medicine and public health, pediatrics, family and community medicine, microbiology, and community nursing, without a unified structure or consistent depth (17–21). Thus, students’ or residents’ exposure to the field largely depends on their personal interest or the initiative of individual faculty members.

At the postgraduate level, the gap is even more evident: the demand for advanced vaccinology training substantially exceeds the available capacity. In 2022, out of 33 advanced vaccinology courses worldwide, only 5 were at the master’s level (4). Currently, there are 38 advanced courses, of which only 8 confer a master’s degree (1). The majority of these courses are not discipline-specific and include vaccine-related modules embedded in broader programs. In Spain, there are no consolidated official master’s degrees that cover the full vaccine cycle from translational research through program implementation. Instead, training relies primarily on non-official master’s degrees (Spanish *título propio*), which vary widely in quality, structure, and scope.

Spanish postgraduate programs in vaccinology have adopted a multidisciplinary orientation, admitting graduates from diverse health and biomedical backgrounds. This openness reflects the field’s inherent transversality, spanning foundational immunology and specialized competencies, including vaccine administration techniques, clinical trial design, and regulatory management.

The international literature consistently calls for the establishment of minimum competency standards to ensure that graduates are prepared to contribute to the design, evaluation, and management of safe and efficient immunization strategies. A recent study by Llimós et al. on Spanish master’s programs in public health described a similarly fragmented curriculum and argued for the development of a national core curriculum (22).

The primary objective of the present study was to systematically map and analyze the academic characteristics and curricular content of Spanish master’s-level programs in vaccinology, identifying convergences, gaps, and opportunities for improvement in relation to international standards. The secondary objective was to propose a Delphi-informed, consensus-based core curriculum for a master’s degree in vaccinology.

## 2 Methods

### 2.1 Study design and protocol

We conducted a nationwide systematic environmental scan of Spanish master’s-level programs relevant to vaccinology, followed by a modified Delphi to propose method to propose a competency-based core curriculum. A prospectively specified protocol defined data sources, inclusion criteria, duplicate screening and extraction procedures, and Delphi procedures. The search strategy was peer-reviewed by independent experts to ensure its completeness.

### 2.2 Data sources and search strategy

Between May and June 2025, we searched (i) the Registro de Universidades, Centros y Títulos (RUCT); (ii) the degree search portal of ANECA; and (iii) the institutional portals/websites of 80 Spanish universities and relevant professional schools. Bilingual keywords (Spanish/English) covered vaccinology, immunization, vaccines, and related public-health terms. We documented the sources consulted, search strings, dates, and filters used; records from different sources were consolidated and deduplicated. To transparently document the identification and selection process, we present a Preferred Reporting Items for Systematic Reviews and Meta-Analyses (PRISMA)-style flow diagram adapted for environmental scans.

### 2.3 Eligibility criteria

We included master’s-level programs that were active during the academic year 2025–2026, had a publicly accessible official syllabus, and allocated at least 20 European Credit Transfer and Accumulation System (ECTS) credits specifically to vaccinology or immunization (e.g., program aims or ≥1 compulsory module primarily focused on vaccines/immunization). Degree type was classified as official master’s degree (state-regulated, RUCT-listed/ANECA-accredited) and non-official master’s degree (“título propio,” created and accredited internally by the university under its institutional autonomy and not recognized as an official degree in the national framework). We excluded short courses, diplomas/certificates without ECTS equivalence, and inactive or undocumented offerings.

### 2.4 Screening, selection, and inter-rater reliability

Two reviewers independently screened program titles/webpages and assessed full syllabi against pre-specified eligibility criteria; reasons for exclusion were recorded, and disagreements were resolved by consensus. A PRISMA-style diagram summarizes the identification, screening, eligibility, and inclusion.

Inter-rater reliability was evaluated in two phases: (i) title/abstract screening of deduplicated programs and (ii) full-text eligibility. In both phases, the reviewers worked in duplicate and were blinded to each other’s decisions. We computed percent agreement (Po) and Cohen’s kappa (*κ*), with κ ≥ 0.60 considered acceptable *a priori*.

### 2.5 Data extraction and variables

Using a piloted template, two reviewers independently extracted the university/organizing body, degree type (official vs. non-official), total ECTS, delivery format (on-site/online/hybrid), language of instruction, tuition (EUR; EU vs. non-EU, where available), admission profile, practicum/internship (presence and ECTS), and master’s thesis requirement. Mandatory curricular content was mapped to predefined thematic domains.

These thematic domains were developed a *priori* through an iterative process. We first drafted a provisional list informed by previous descriptions of advanced vaccinology programs and public-health competency frameworks, then piloted it on two representative syllabi. Two reviewers independently mapped all mandatory modules to the provisional domains, compared their classifications, and refined the domains by consensus. This final framework was used to code all programs, assigning each compulsory module to the domain that best reflected its primary learning objective; the domains in Table 1 correspond to this framework.

**Table 1:** Mandatory content and number of ECTS (European Credit Transfer and Accumulation System) in Spanish master’s programs in vaccinology (2025–2026).

Curricular domain	Máster Erasmus Mundus (LIVE)* (23)	Máster^1^ EPA (24)	Máster en Inmunoterapia y Vacunas (25)	Máster en Investigación Inmunoterapia y vacunas^2^ (26)	Máster en Vacunas en enfermería (27)	Máster en Vacunación^3^ (28)	Máster Experto en Vacunas (EC–VAC) (29)
Scientific fundamentals I: immunology, microbiology, virology, parasitology.	Yes (24 ECTS)	Yes (ECTS N/R)	Yes (6 ECTS)	Yes (6 ECTS)	Yes (ECTS N/R)	Yes (ECTS N/R)	Yes (ECTS N/R)
Scientific fundamentals II: basic vaccinology, vaccine-preventable diseases, immunodeficiencies, immuno-oncology, and autoimmunity.	Yes (21 ECTS)	Yes (ECTS N/R)	Yes (6 ECTS)	Yes (12 ECTS)	Yes (ECTS N/R)	Yes (ECTS N/R)	Yes (ECTS N/R)
Development, production, and pharmacovigilance in vaccinology I: development and production.	Yes (3 ECTS)	Yes (ECTS N/R)	Yes (6 ECTS)	Yes (6 ECTS)	Yes (ECTS N/R)	Yes (ECTS N/R)	Yes (ECTS N/R)
Development, production, and pharmacovigilance in vaccinology II: regulation and pharmacovigilance.	Yes (3 ECTS)	No	Yes (12 ECTS)	Yes (6 ECTS)	Yes (ECTS N/R)	Yes (ECTS N/R)	Yes (ECTS N/R)
Epidemiology and public health	Yes (9 ECTS)	Yes (ECTS N/R)	Yes (6 ECTS)	Yes (6 ECTS)	Yes (ECTS N/R)	Yes (ECTS N/R)	Yes (ECTS N/R)
Cross–cutting competencies: program management, leadership, and scientific communication.	Yes (3 ECTS)	Yes (ECTS N/R)	No	Yes (6 ECTS)	Yes (ECTS N/R)	Yes (ECTS N/R)	Yes (ECTS N/R)
Vaccination in special populations	Yes (3 ECTS)	No	No	No	Yes (ECTS N/R)	Yes (ECTS N/R)	Yes (ECTS N/R)
Translational and applied research	Yes (12 ECTS)	Yes (ECTS N/R)	Yes (6 ECTS)	Yes (6 ECTS)	Yes (ECTS N/R)	Yes (ECTS N/R)	Yes (ECTS N/R)
Master’s Thesis (TFM)	Yes 27 ECTS	Yes (ECTS N/R)	Yes 12 ECTS	Yes (12 ECTS)	No	No	No
Professional internships	Yes (3 ECTS)	No	No	No	No	No	No

N/R, not reported (ECTS not specified in the official syllabus). The remaining 12 ECTS correspond to foreign-language coursework.

1 UCAM–EPA.

2 UNIR–NG.

3 ESNECA.

### 2.6 Handling of costs and missing data

Tuition was recorded in EUR with the month/year of extraction noted. When portals displayed discounts or ranges, we recorded the base listed tuition. When information was unclear or unavailable, archived pages or program contacts were consulted; unresolved items were marked not reported (N/R) and flagged in table notes.

### 2.7 Delphi consensus

A modified Delphi was conducted with five experts in preventive medicine and vaccinology to propose a core curriculum and to rate the essentiality of items on a 5-point Likert scale. The initial list of domains assessed in the Delphi process was derived from the thematic patterns and gaps identified during the environmental scan, ensuring that the curriculum design was grounded in empirical findings. Consensus was prespecified as a mean ≥4 with ≥80% of ratings in categories 4–5. Items without consensus were revised or excluded between the two rounds; stability across rounds and response rates are reported in the supplement.

### 2.8 Ethics

The environmental scan relied on publicly available information and did not involve human participants. Delphi panelists were adult professionals who provided informed consent to participate.

## 3 Results

**Figure 1:**
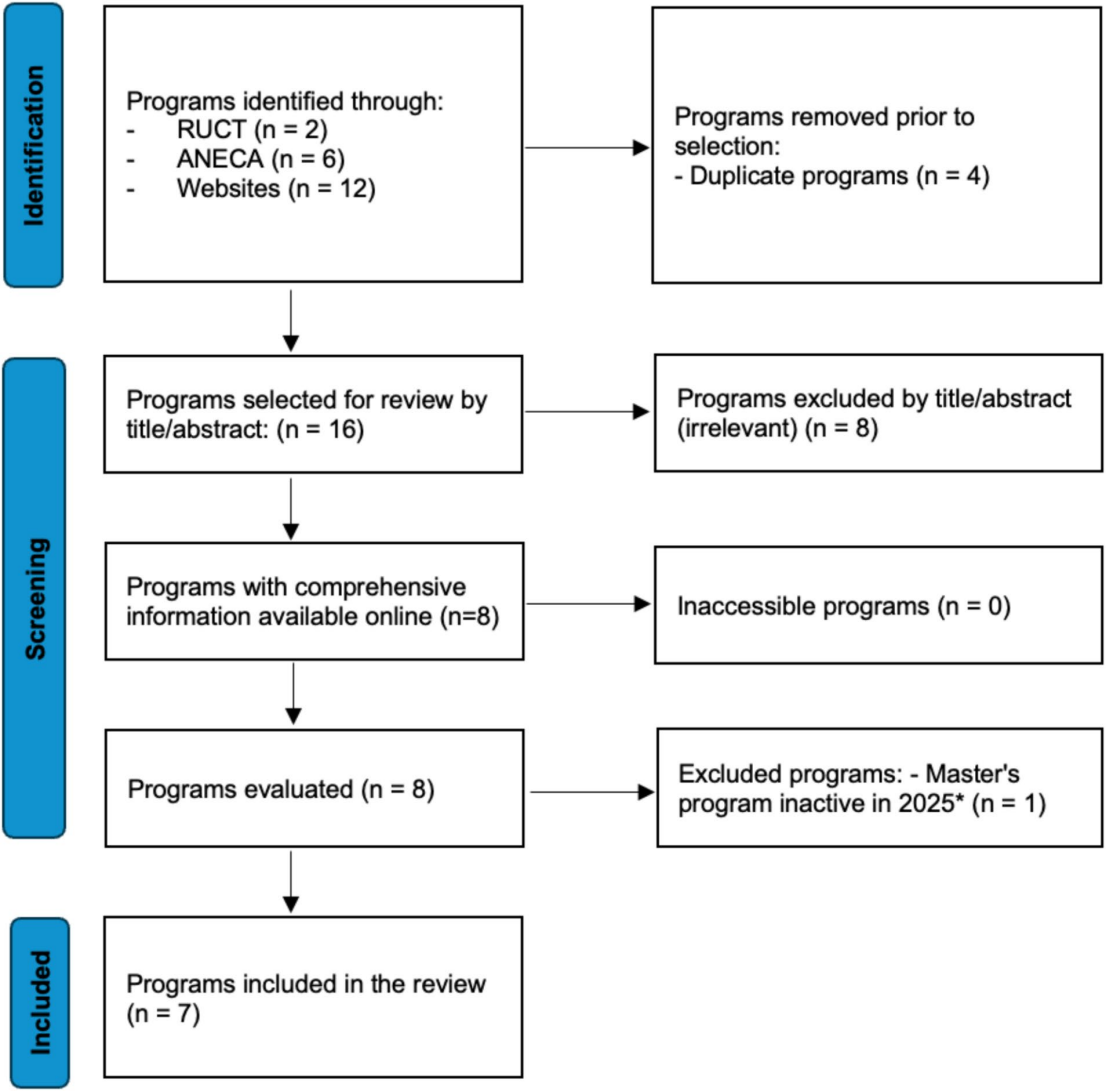
PRISMA-style flow diagram summarizing identification, screening, eligibility, and inclusion, combining registry and other web sources.

Twenty master’s programs were identified across sources: RUCT (*n* = 2), ANECA (*n* = 6), and institutional websites (*n* = 12). Seven programs met eligibility criteria and were included in the synthesis (Figure 1; Supplementary material Annex 2).

Inter-rater reliability was acceptable across phases. During title/abstract screening (*n* = 16), observed agreement was 93.75% with a Cohen’s *κ* of 0.875. During full-text eligibility assessment (*n* = 8), agreement was 100% with κ = 1.00. Both phases exceeded the prespecified threshold (κ ≥ 0.60), indicating good to excellent reviewer consistency.

The analysis identified seven programs related to vaccinology or adjacent areas (23–29). The Erasmus Mundus LIVE (Leading International Vaccinology Education) Master’s Degree was the only program awarding an official master’s degree and the only one with 120 European Credit Transfer and Accumulation System (ECTS) credits. LIVE was also the only program with an explicitly international, multi-institutional organizational focus; the remaining programs were nationally oriented. Delivery was predominantly online (86%). The language of instruction was English for LIVE and Spanish for the remaining programs. Four programs required a master’s thesis: LIVE (27 ECTS), Máster en Inmunoterapia y Vacunas (VIU–IV; 12 ECTS), Máster de Formación Permanente en Investigación en Inmunoterapia y Vacunas de Nueva Generación (UNIR–NG; 12 ECTS), and the UCAM–EPA program (ECTS not reported). Only LIVE offered professional internships (3 ECTS). Total tuition ranged from €560 to €10,159, with the lowest cost for the Máster Experto en Vacunas (EC–VAC) and the highest for non-EU applicants to LIVE (Table 2).

**Table 2:** Academic characteristics of postgraduate programs in vaccinology/vaccines in Spain (academic year 2025–2026).

Program title	Máster Erasmus Mundus (LIVE)* (23)	Máster EPA^1^ (24)	Máster en Inmunoterapia y Vacunas (25)	Máster en Investigación en Inmunoterapias y vacunas^2^ (26)	Máster en Vacunas en enfermería (27)	Máster en Vacunación^3^ (28)	Máster Experto en Vacunas (EC–VAC) (29)
University	UAB – Spain UB – Spain UAntwerpen – Belgium UJM – France UCBL – France	UCAM – Univ. Católica de Murcia	Universidad Internac. de Valencia (VIU)	Universidad Internac. de La Rioja (UNIR)	TECH Universidad Tecnológica	ESNECA Business School	Escuela Clínica y Ciencias de la Salud (ECCS)
Official degree	Yes	No	No	No	No	No	No
ECTS	120	60	60	60	60	24*	30*
Delivery format	On-site (2 years)	Online (synchronous classes)	Online	Interactive online	Online (7 months)	Online	Online
Language	English	Spanish	Spanish	Spanish	Spanish	Spanish	Spanish
Seats	30 seats	50 seats	“Limited seats”	N/R	N/R	N/R	N/R
Master’s thesis (ECTS)	Yes (27 ECTS)	Yes (ECTS not specified)	Yes (12 ECTS)	Yes (12 ECTS)	No	No	No
Professional internships (ECTS)	(Integrated practical training in the 4th semester, 3 ECTS)	No	No	No	No	No	No
Total tuition (€)	**€2,646** - (€10,159 for non-EU citizens)	**2,100€**	6,000€–3,492€ (discount)	**Not disclosed**	**3,900€**	3,460€–**1,120€** (discount)	2,240€–**560€** (discount)
Admission profile	^4^Health Sciences, Bioengineers.	Nursing	^4^Health Sciences, Bioengineering, Bioinformatics, Biomedicine.	^4^Health Sciences, Biomedicine, Biotechnology.	Nursing	Not disclosed	^4^Health Sciences

1 UCAM–EPA = Máster en Enfermería de Práctica Avanzada en Inmunizaciones.

2 UNIR–NG = Máster de Formación Permanente en Investigación en Inmunoterapia y Vacunas de Nueva Generación.

3 ESNECA = Máster en Epidemiología y Salud Pública + Máster en Vacunación.

4 Admission profiles typically include Medicine, Nursing, Pharmacy, Biology, Veterinary Medicine, and Biochemistry.

* Programs that offer a certificate with ECTS equivalence.

### 3.1 Admission profile

Admission profiles spanned multiple health sciences degrees. The UCAM–EPA and TECH–VE (Máster en Vacunas en Enfermería) programs targeted nursing professionals; the ESNECA program (Máster en Epidemiología y Salud Pública + Máster en Vacunación) did not disclose an admission profile; the remaining programs were open to a variety of health-related backgrounds (Table 2).

## 4 ECTS by curricular domains

ECTS assigned to each curricular domain were available for LIVE, VIU–IV, and UNIR–NG; the remaining programs did not report domain-level ECTS (Table 1).

All master’s programs included modules on scientific foundations of vaccinology, vaccine development and safety I (development/production), epidemiology and public health, and translational/applied research. Most programs (86%) delivered mandatory content on vaccine development and safety II (regulation and pharmacovigilance), and 57% addressed cross-cutting competencies (management, leadership, and scientific communication), as summarized in Table 1 (and visually in Supplementary material Annex 1).

### 4.1 Selection of vaccinology curricular domains for inclusion in master’s programs

Experts rated the identified content using a Likert scale (1–4, 6) and provided qualitative comments. After two rounds, consensus on the final domain was defined as a mean score ≥4 with ≥80% agreement across participants. These domains, together with their proposed ECTS allocations, constitute a 60-ECTS core curriculum for master’s-level training in vaccinology (Table 3). The initial set of domains submitted to the Delphi evaluation was derived from the thematic patterns and gaps identified during the environmental scan, ensuring that the curriculum design was directly informed by empirical findings.

**Table 3:** Delphi results for the selection of curricular domains in a national master’s degree in vaccinology.

Curricular domain	Average score (Likert 1–5)	Consensus reached (%)	Proposed ECTS credits	Main content
Scientific fundamentals	4.8	100%	6	Basic and clinical immunology, microbiology, virology, pathogenesis of vaccine-preventable diseases, autoimmunity, immuno-oncology, and immunodeficiencies.
Development, Production, and Pharmacovigilance in Vaccinology	4.6	100%	6	Vaccine development cycle (preclinical, clinical phases, post-marketing), innovative platforms (mRNA, viral vectors, protein subunits), quality control, industrial manufacturing, EMA/FDA/WHO regulatory aspects, pharmacovigilance, and vaccine safety.
Epidemiology and Public Health	4.9	100%	12	Basic and advanced epidemiology, vaccine program design and evaluation, health economics, One Health, climate change, pandemic preparedness and response, epidemiological surveillance, population immunization strategies, and impact assessment.
Cross-Cutting competencies	4.5	80%	6	Management and leadership of vaccination programs, scientific and health communication, counseling and motivational interviewing, health literacy, ethical and social aspects of vaccination, and combating misinformation.
Vaccination in special populations	4.3	80%	6	Vaccination in pregnant women, immunocompromised patients (transplants, HIV, chemotherapy, immunosuppressants), autoimmune diseases, international travelers, the older adults, and other at-risk groups.
Translational and Applied Research	4.4	80%	6	Clinical trials and immunogenicity, clinical and epidemiological study design, applied bioinformatics, effectiveness and safety data analysis, development of new vaccine platforms, implementation and evaluation research.
Master’s Thesis (TFM)	4.7	100%	12	Individual research, innovation, or vaccine implementation project; includes hypothesis formulation, methodology, results analysis, and defense before a panel.
Professional internships	4.2	80%	6	Clinical rotations in hospital or public health vaccination services, the pharmaceutical industry, regulatory agencies, or international institutions (WHO, EMA, ministries of health). Practical application of skills in real-world settings.

## 5 Discussion

Postgraduate training in vaccinology in Spain largely occurs outside the official university system. Most instruction is delivered online, and programs exhibit a heterogeneous modular structure. Although all programs share immunologic and clinical fundamentals, notable gaps persist.

Only four programs allocate substantive credits to vaccination in special/risk populations, despite these populations being among the fastest-growing areas of vaccinology and operating within a rapidly evolving regulatory landscape at both the national and regional (Autonomous Communities) levels. This is an area that should be addressed by all vaccinology programs delivered in Spain (30–32).

When compared with established international programs, including the MSc in Vaccinology and Drug Development (University of Siena) (33), and the Infectious Diseases and Vaccinology MPH at UC Berkeley (34), the MSc (Med) in Vaccinology (Wits University) (35), Spanish programs display greater heterogeneity in curricular depth, fewer credits devoted to regulatory science and health economics, and minimal incorporation of internships or structured research experiences.

Another gap identified is that most programs are non-official master’s degrees and are not required to include a Master’s thesis (TFM). Three of the analyzed programs do not include a thesis, thereby missing the opportunity for students to apply their learning in a capstone project. Requiring a thesis can generate constructive feedback loops and scientific enrichment for programs while fostering research output in vaccinology.

These findings indicate heterogeneity and notable gaps across Spanish master’s programs in vaccinology. The absence of a minimum competency framework (comparable, for instance, to the ASPHER list for public health professionals) hampers comparability and European professional mobility (36). We therefore propose that professional societies promote a common 60-ECTS core curriculum, to include:

### 5.1 Scientific fundamentals: 6 ECTS

In Spanish programs, this domain is generally present, though with variable depth (23, 33–35, 37). These competencies are essential to understand immune responses to vaccination, mechanisms of protection, and determinants of effectiveness and safety. Including modules on pathogenesis and immunopathology links basic biology to clinical practice and underpins the remaining curricular domains.

### 5.2 Vaccine development and production: 6 ECTS

The vaccine development cycle is broadly covered in Spanish programs. Training in industrial production, quality, and process control ensures that graduates understand manufacturing challenges, while exposure to new technological platforms prepares them for a rapidly evolving field (23, 33–35, 37).

### 5.3 Epidemiology and public health: 12 ECTS

All Spanish master’s programs include epidemiology and public health; however, not all integrate Health Economics, health, climate change, and pandemic preparedness, which are essential to impact assessment and vaccination policy decision-making (23, 33, 35). International programs tend to cover these topics more extensively, making them central to the curriculum (23, 34, 37).

### 5.4 Cross-cutting competencies: 6 ECTS

These competencies enable graduates not only to generate knowledge but also to lead immunization programs, communicate effectively with diverse audiences, and respond to societal challenges such as vaccine misinformation. Ethics, equity, and social dimensions should be addressed throughout the curriculum, consistent with their growing relevance in global health (23, 35, 37).

### 5.5 Vaccination in special populations: 6 ECTS

Patients at increased risk of infection, often with suboptimal immune responses or contraindications to live vaccines, require a differentiated, up-to-date clinical approach. Furthermore, international travel vaccination is essential for individual prevention and global epidemiological control (30, 38). Given the dynamic regulatory context and the expanding evidence base, it is indispensable that an official master’s in vaccinology incorporate systematic modules on special populations to ensure comprehensive training aligned with current public-health challenges (39).

### 5.6 Translational and applied research: 6 ECTS

This domain prepares students in clinical and epidemiologic research methods and in the use of bioinformatic tools for data analysis. Translational vaccinology is operationalized through modules on immunogenicity trials, clinical study design, and evaluation of novel vaccine platforms (23, 35, 37).

### 5.7 Master’s thesis (TFM): 12 ECTS

Although some national programs do not include a thesis, students should have the opportunity to apply their learning in a substantive final project. A 12-ECTS thesis consolidates research experience, fosters critical thinking, and supports the generation of actionable knowledge for public health, clinical practice, and vaccine innovation, aligning with international master’s programs (23, 35, 37).

### 5.8 Professional internships: 6 ECTS

A further relevant finding is the scarcity of professional internships in Spanish programs. Most do not offer rotations in healthcare institutions or industry—unlike many international official programs, where internships are compulsory and provide an interface between academia and institutions such as the WHO, regulatory agencies (e.g., EMA), or pharmaceutical companies (23, 33–35, 37).

This study thus shows marked heterogeneity across Spanish programs in vaccinology, including degree status (official vs. non-official), admission profiles, total tuition, research weight, coverage of special populations, availability of internships, and total duration. While there are accessible options tailored to different professional backgrounds, Spanish programs still fall short of international standards in training load, integration with practice, and research opportunities.

This study relied on publicly available information from official registries and institutional websites, which may not fully capture recent curricular changes or program implementation. Moreover, data on faculty qualifications and industry participation were not systematically accessible. Future research should address these aspects as key indicators of academic rigor and educational quality.

Overall, the analysis underscores the need to move toward a national core curriculum in vaccinology that ensures solid, homogeneous, and internationally comparable training. The lack of professional internships and the non-mandatory nature of the thesis (TFM) in some programs limit graduates’ ability to integrate into research teams and healthcare institutions.

## 6 Conclusion

It is a priority for universities, together with professional societies and regulatory agencies, to advance an official master’s program in vaccinology with a common core of at least 60 ECTS, including both theoretical training and applied research, thereby ensuring international comparability and quality of training.

## Data Availability

The raw data supporting the conclusions of this article will be made available by the authors, without undue reservation.

## Footnotes

1 https://www.icavt.org/
