# Supplementary material for: Master’s programs in vaccinology in Spain: a nationwide systematic environmental scan and a Delphi-informed core curriculum proposal
Source: Front Public Health. 2026 Feb 2;14:1707015. doi: 10.3389/fpubh.2026.1707015 (PMC12908584; doi:10.3389/fpubh.2026.1707015)
Supplement: Supplementary file 1 [file Data_Sheet_1.docx]

# Supplementary Material:

## Annex 1. Coverage of mandatory content across Spanish vaccinology programs (academic year 2025–2026; n=7). Labels show the percentage of programs including each domain. Source: RUCT, ANECA, and institutional portals (May–June 2025).

## Annex 2:

Search strategy on RUCT, ANECA, and website platforms.

| **Platform** | **Results** | **Excluded?** | **Reason for exclusion** | **Website** |
| --- | --- | --- | --- | --- |
| **RUCT** | 2 | 1 | Duplicated | <https://www.educacion.gob.es/ruct/consultaestudios?actual=estudios> |
| **Criteria:** “title name” no other exclusion criteria were used. It does not use a thesaurus **Search Terms:** vacuna, vacunas, vacunología, vacunologia, inmunización, inmunización e inmunoterapia |  |  |  |  |
|  |  |  |  |  |
|  |  |  |  |  |
|  |  |  |  |  |
|  |  |  |  |  |
|  |  |  |  |  |
| **ANECA** | 6 | 6 | “Not relevant (unrelated to vaccines)” | <https://srv.aneca.es/ListadoTitulos/> |
| **Criteria:** The website has a free-text title search engine; it does not use a thesaurus.  **Search Terms:** vacuna, vacunas, vacunología, vacunologia, inmunización, inmunización e inmunoterapia |  |  |  |  |
|  |  |  |  |  |
|  |  |  |  |  |
|  |  |  |  |  |
|  |  |  |  |  |
|  |  |  |  |  |
| **Universities and professional schools** |  |  |  |  |
| Universidad de Alcalá (UAH) | 1 | 1 | “Not relevant (unrelated to vaccines)” | https://www.uah.es |
| Universidad de Alicante (UA) | 0 | 0 |  | https://www.ua.es |
| Universidad de Almería (UAL) | 0 | 0 |  | https://www.ual.es |
| Universitat Autònoma de Barcelona (UAB) | 1 | 1 | Duplicated | https://www.uab.cat |
| Universidad Autónoma de Madrid (UAM) | 0 | 0 |  | https://www.uam.es |
| Universitat de Barcelona (UB) | 1 | 1 | Duplicated | https://www.ub.edu |
| Universidad de Burgos (UBU) | 0 | 0 |  | https://www.ubu.es |
| Universidad de Cádiz (UCA) | 0 | 0 |  | https://www.uca.es |
| Universidad de Cantabria (UNICAN) | 0 | 0 |  | https://www.unican.es |
| Universidad Carlos III de Madrid (UC3M) | 0 | 0 |  | https://www.uc3m.es |
| Universidad de Castilla-La Mancha (UCLM) | 0 | 0 |  | https://www.uclm.es |
| Universidad Complutense de Madrid (UCM) | 0 | 0 |  | https://www.ucm.es |
| Universidad de Córdoba (UCO) | 0 | 0 |  | https://www.uco.es |
| Universidade da Coruña (UDC) | 0 | 0 |  | https://www.udc.gal |
| Universidad de Extremadura (UNEX) | 0 | 0 |  | https://www.unex.es |
| Universitat de Girona (UdG) | 0 | 0 |  | https://www.udg.edu |
| Universidad de Granada (UGR) | 0 | 0 |  | https://www.ugr.es |
| Universidad de Huelva (UHU) | 0 | 0 |  | https://www.uhu.es |
| Universidad Internacional de Valencia (VIU) | 1 | 0 |  | <https://www.universidadviu.com/es/master-inmunoterapia-vacunas> |
| Universitat de les Illes Balears (UIB) | 0 | 0 |  | https://www.uib.es |
| Universidad de Jaén (UJA) | 0 | 0 |  | https://www.ujaen.es |
| Universidad de La Laguna (ULL) | 0 | 0 |  | https://www.ull.es |
| Universidad de La Rioja (UR) | 0 | 0 |  | https://www.unirioja.es |
| Universidad de Las Palmas de Gran Canaria (ULPGC) | 0 | 0 |  | https://www.ulpgc.es |
| Universidad de León (ULE) | 0 | 0 |  | https://www.unileon.es |
| Universitat de Lleida (UdL) | 0 | 0 |  | https://www.udl.cat |
| Universidad de Málaga (UMA) | 0 | 0 |  | https://www.uma.es |
| Universidad Miguel Hernández de Elche (UMH) | 0 | 0 |  | https://www.umh.es |
| Universidad de Murcia (UM) | 0 | 0 |  | https://www.um.es |
| Universidad Pública de Navarra (UPNA) | 0 | 0 |  | https://www.unavarra.es |
| Universidad de Oviedo (UNIOVI) | 0 | 0 |  | https://www.uniovi.es |
| Universidad del País Vasco / Euskal Herriko Unibertsitatea (UPV/EHU) | 0 | 0 |  | https://www.ehu.eus |
| Universidad Politécnica de Cartagena (UPCT) | 0 | 0 |  | https://www.upct.es |
| Universitat Politècnica de Catalunya (UPC) | 0 | 0 |  | https://www.upc.edu |
| Universitat Politècnica de València (UPV) | 0 | 0 |  | https://www.upv.es |
| Universidad Politécnica de Madrid (UPM) | 0 | 0 |  | https://www.upm.es |
| Universitat Pompeu Fabra (UPF) | 0 | 0 |  | https://www.upf.edu |
| Universidad Rey Juan Carlos (URJC) | 0 | 0 |  | https://www.urjc.es |
| Universitat Rovira i Virgili (URV) | 0 | 0 |  | https://www.urv.cat |
| Universidad de Salamanca (USAL) | 0 | 0 |  | https://www.usal.es |
| Universidade de Santiago de Compostela (USC) | 0 | 0 |  | https://www.usc.gal |
| Universidad de Sevilla (US) | 1 | 1 | Inactive program | https://www.us.es |
| Universitat de València (UV) | 0 | 0 |  | https://www.uv.es |
| Universidad de Valladolid (UVA) | 0 | 0 |  | https://www.uva.es |
| Universidade de Vigo (UVigo) | 0 | 0 |  | https://www.uvigo.gal |
| Universidad de Zaragoza (UNIZAR) | 1 | 1 | Not relevant (unrelated to vaccines) | https://www.unizar.es |
| Universitat Jaume I (UJI) | 0 | 0 |  | https://www.uji.es |
| Universidad Internacional Menéndez Pelayo (UIMP) | 1 | 1 | Not relevant (unrelated to vaccines) | https://www.uimp.es |
| Universidad Pablo de Olavide (UPO) | 0 | 0 |  | https://www.upo.es |
| Universidad Internacional de Andalucía (UNIA) | 0 | 0 |  | https://www.unia.es |
| Universidad Nacional de Educación a Distancia (UNED) | 0 | 0 |  | https://www.uned.es |
| Universidad de Navarra (UNAV) | 0 | 0 |  | https://www.unav.edu |
| Universidad Pontificia Comillas | 0 | 0 |  | https://www.comillas.edu |
| Universidad Pontificia de Salamanca (UPSA) | 0 | 0 |  | https://www.upsa.es |
| Universidad de Deusto | 0 | 0 |  | https://www.deusto.es |
| Universidad CEU San Pablo | 0 | 0 |  | https://www.uspceu.com |
| Universidad CEU Cardenal Herrera (UCH-CEU) | 0 | 0 |  | https://www.uchceu.es |
| Universitat Abat Oliba CEU (UAO) | 0 | 0 |  | https://www.uaoceu.es |
| Universitat Ramon Llull (URL) | 0 | 0 |  | https://www.url.edu |
| Universitat Internacional de Catalunya (UIC Barcelona) | 0 | 0 |  | https://www.uic.es |
| Universitat Oberta de Catalunya (UOC) | 0 | 0 |  | https://www.uoc.edu |
| Universidad Internacional de La Rioja (UNIR) | 1 | 0 |  | https://www.unir.net |
| Universidad de Mondragón (MU) | 0 | 0 |  | https://www.mondragon.edu |
| Universidad San Jorge (USJ) | 0 | 0 |  | https://www.usj.es |
| Universidad Francisco de Vitoria (UFV) | 0 | 0 |  | https://www.ufv.es |
| Universidad Camilo José Cela (UCJC) | 0 | 0 |  | https://www.ucjc.edu |
| Universidad Antonio de Nebrija (Nebrija) | 0 | 0 |  | https://www.nebrija.com |
| Universidad TECH institute | 0 | 0 |  | https://www.techtitute.com/enfermeria/master/master-vacunas-enfermeria |
| Universidad Alfonso X el Sabio (UAX) | 0 | 0 |  | https://www.uax.com |
| Universidad Europea de Madrid (UEM) | 0 | 0 |  | https://universidadeuropea.com |
| Universidad Europea de Valencia (UEV) | 0 | 0 |  | https://universidadeuropea.com |
| Universidad Europea de Canarias (UEC) | 0 | 0 |  | [https://universidadeuropea.com](https://www.uah.es/) |
| Universidad a Distancia de Madrid (UDIMA) | 0 | 0 |  | https://www.udima.es |
| Universidad Católica de Valencia San Vicente Mártir (UCV) | 0 | 0 |  | https://www.ucv.es |
| Universidad Católica San Antonio de Murcia (UCAM) | 1 | 0 |  | https://www.ucam.edu |
| Universidad Isabel I (UI1) | 0 | 0 |  | https://www.ui1.es |
| IE University | 0 | 0 |  | https://www.ie.edu/university/ |
| ESNECA Business School | 1 | 0 |  | <https://www.esneca.com/formacion/master-vacunacion-epidemiologia/> |
| TECH Universidad Tecnológica | 1 | 0 |  | https://www.techtitute.com/ |
| Escuela Clínica y de Ciencias de la Salud | 1 | 0 |  | <https://escuelaclinica.com/cursos/master-experto-vacunas/?tab=tab-overview> |
